# Evaluation of White Cell Count and Differential in Synovial Fluid for Diagnosing Infections after Total Hip or Knee Arthroplasty

**DOI:** 10.1371/journal.pone.0084751

**Published:** 2014-01-08

**Authors:** Xinhua Qu, Zanjing Zhai, Xuqiang Liu, Haowei Li, Chuanlong Wu, Yang Li, Huiwu Li, Zhenan Zhu, An Qin, Kerong Dai

**Affiliations:** Department of Orthopedics, Shanghai Key Laboratory of Orthopedic Implant, Shanghai Ninth People’s Hospital, Shanghai Jiaotong University School of Medicine, Shanghai, China; IPO, Inst Port Oncology, Portugal

## Abstract

**Background:**

The accuracy of synovial fluid (SF) white cell count (WCC) and polymorphonuclear (PMN) cell evaluation for predicting prosthetic joint infection (PJI) at the total hip arthroplasty (THA) or total knee arthroplasty (TKA) site is unknown. Therefore, we performed a meta-analysis to summarize the diagnostic validity of SF-WCC and SF-PMN for diagnosing PJI.

**Methods:**

The MEDLINE, EMBASE, and OVID databases were searched for studies that had evaluated the diagnostic validity of SF-WCC and SF-PMN between January 1990 and May 2013. Meta-analysis methods were used to pool sensitivity, specificity, diagnostic odd ratios (DORs), the area under the receiver-operating characteristic curve (AUC), positive likelihood ratios (PLR), negative likelihood ratios (NLR), and post-test probability. We also conducted heterogeneity, publication bias, subgroup, and meta-regression analyses.

**Results:**

Fifteen articles (15 SF-WCC and 14 SF-PMN) that included a total of 2787 patients fulfilled the inclusion criteria and were considered for analysis. The pooled sensitivity and specificity for PJI detection was 0.88 (95% confidence intervals [CI], 0.81–0.93) and 0.93 (95% CI, 0.88–0.96) for SF-WCC and 0.90 (95% CI, 0.84–0.93) and 0.88 (95% CI, 0.83–0.92) for SF-PMN, respectively. The AUC was 0.96 for SF-WCC and 0.95 for SF-PMN. PLR and NLR were 13.3 and 0.13 for SF-WCC, and 7.6 and 0.12 for SF-PMN, respectively. There was no evidence of publication bias. Low-clinical-scenario (pre-test probability, 20%) post-test probabilities were 3% for both negative SF-WCC and SF-PMN results. The subgroup analyses indicated that the sensitivity/specificity of THA were 0.73/0.96 for SF-WCC and 0.85/0.83 for SF-PMN, whereas those of TKA were 0.90/0.91 for SF-WCC and 0.90/0.88 for SF-PMN. We also found that collection of SF-WCC preoperatively had a higher sensitivity than that obtained intraoperatively (0.91 vs. 0.77).

**Conclusions:**

SF-WCC and SF-PMN have an adequate and clinically acceptable diagnostic value for detecting PJI, particularly after TKA.

## Introduction

Prosthetic joint infection (PJI) is one of the most common complications of total hip arthroplasty (THA) and total knee arthroplasty (TKA) that occurs in 1–12% surgical cases and is associated with a number of adverse outcomes [1], [2]. A multitude of tests have been developed for diagnosing PJI, including preoperative laboratory testing, radiological examination, nuclear medicine detection, intraoperative culture, and histopathology [3]. However, there is no established gold standard test for diagnosing PJI, and the limited sensitivity and specificity of the available tests make it difficult to distinguish between PJI and other causes of prosthetic failure, such as metal allergy or aseptic loosening [2], [4].

Synovial fluid (SF) white cell count (WCC) and polymorphonuclear (PMN) cell counts, which can be rapidly obtained from preoperative or intraoperative aspiration, and have a faster turnaround-time, may play a role in diagnosis of PJI [5]–[9]. The guidelines of the American Academy of Orthopaedic Surgeons (AAOS) and Infectious Diseases Society of America (IDSA) strongly recommend SF-WCC and SF-PMN for the assessment of PJI [10]–[12]. However, despite the increasing number of publications focused on SF-WCC and SF-PMN for the diagnosis of PJI, the effectiveness of these tests still remains unknown. Therefore, to provide evidence-based advice to physicians on this, we sought to evaluate the detection validity of SF-WCC and SF-PMN for the diagnosis of PJI by using a meta-analysis approach.

## Materials and Methods

The current protocol was performed as recommended by the methodological guidelines for conducting systematic reviews studying diagnostic accuracy [13] and according to the PRISMA statement [14].

### Search Strategy

The MEDLINE, EMBASE, and OVID databases were searched for articles published between January 1990 and May 2013. All searches were performed using the medical subject headings “joint prosthesis,” “prosthesis infection,” “septic loosening,” “aseptic loosening,” “replacement,” and “arthroplasty,” and the free text words “white cell,” “leucocyte,” “PMN,” “polymorphonuclear,” and “synovial fluid”. We did not restrict the search by language. We also manually searched the reference lists of eligible studies and review articles.

### Selection of Studies

Two investigators read the abstracts and used a standardized data extraction form to identify potentially eligible articles. They subsequently read the full text of these articles to determine whether they were eligible for inclusion. Disagreements were resolved by discussing with a third investigator.

The articles required to meet the following qualifications for inclusion in the analysis: (i) collection of data on SF-WCC or SF-PMN along with an accurate diagnosis of PJI as defined by visible purulence of the joint aspirate or at the surgical site, presence of a sinus tract (fistula) communicating with the prosthesis, acute inflammation in histopathology sections of periprosthetic tissue, or simultaneously obtained microbiologic cultures from at least 2 periprosthetic tissue samples (the reference standard); (ii) studies had sufficient data to allow the calculation of the true-positive (TP), false-negative (FN), false-positive (FP), and true-negative (TN) values; and (iii) included ≥10 patients. Discrepancies were resolved by discussing with other investigators and by consulting the original articles.

### Data Extraction and Assessment of Study Quality

Two investigators independently extracted relevant data about the design and results of each study using a standardized form. Observers were not blinded to the journal name, the authors’ names and affiliations, or the year of publication since blinding to these study characteristics has been shown to be unnecessary [15]. To resolve disagreement between reviewers, another reviewer assessed all discrepancies, and the majority opinion was used for the analysis. The methodological quality of the included studies was independently assessed by 2 observers using the QUADAS tool [16], which has been specifically developed for systematic reviews studying diagnostic accuracy.

To perform validity analyses, we extracted the following items from each study using a standardized form: description of study participants, the authors’ names, country where the study was conducted, number of patients, mean age, study design, patient enrolment, the time at which the sample was obtained, exclusion of inflammatory arthropathy, sample type, operative site, the test cut-off, and characteristics of the reference standard used. If a cut-off of >1 was reported, the cut-off values that offered the best test performance were used.

### Statistical Analysis

For each study, we constructed a 2×2 contingency table consisting of TP, FP, FN, and TN results according to the SF-WCC or SF-PMN values and the reference standard. We then calculated the sensitivity as TP/(TP+FN), specificity as TN/(FP+TN), and the diagnostic odds ratios (DOR) as (TP×TN)/(FP×FN). To evaluate the capability of SF-WCC or SF-PMN assays for diagnosing PJI, we estimated the sensitivity, specificity, positive likelihood ratio (PLR), negative likelihood ratio (NLR), DOR, post-test probability, and area under the summary receiver operating characteristic curves (AUC) [17]. Likelihood ratio I^2^ index and χ^2^ tests were used to assess the heterogeneity of the included studies [18]. The I^2^ index is a measure of the percentage of total variation across studies due to heterogeneity. If I^2^ is >50%, it suggests more heterogeneity between studies than that expected by chance alone^10^. For the likelihood ratio χ^2^ test, all p-values <0.05 were considered to indicate heterogeneity between studies. If heterogeneity existed, a random effects model was used for the primary meta-analysis to obtain a summary estimate for the test sensitivity with 95% confidence intervals (CI). We performed meta-regression analyses to assess potential heterogeneity and constructed a Deeks’ funnel plot asymmetry test to evaluate potential publication bias [19]. Subgroup analyses were performed to evaluate different study characteristics (i.e., number of patients, study design, patient enrolment, the time at which the sample was obtained, exclusion of inflammatory arthropathy, and operative site). All the statistical analyses were performed using STATA version 12 (StataCorp, College Station, TX, USA).

## Results

**Figure 1:**
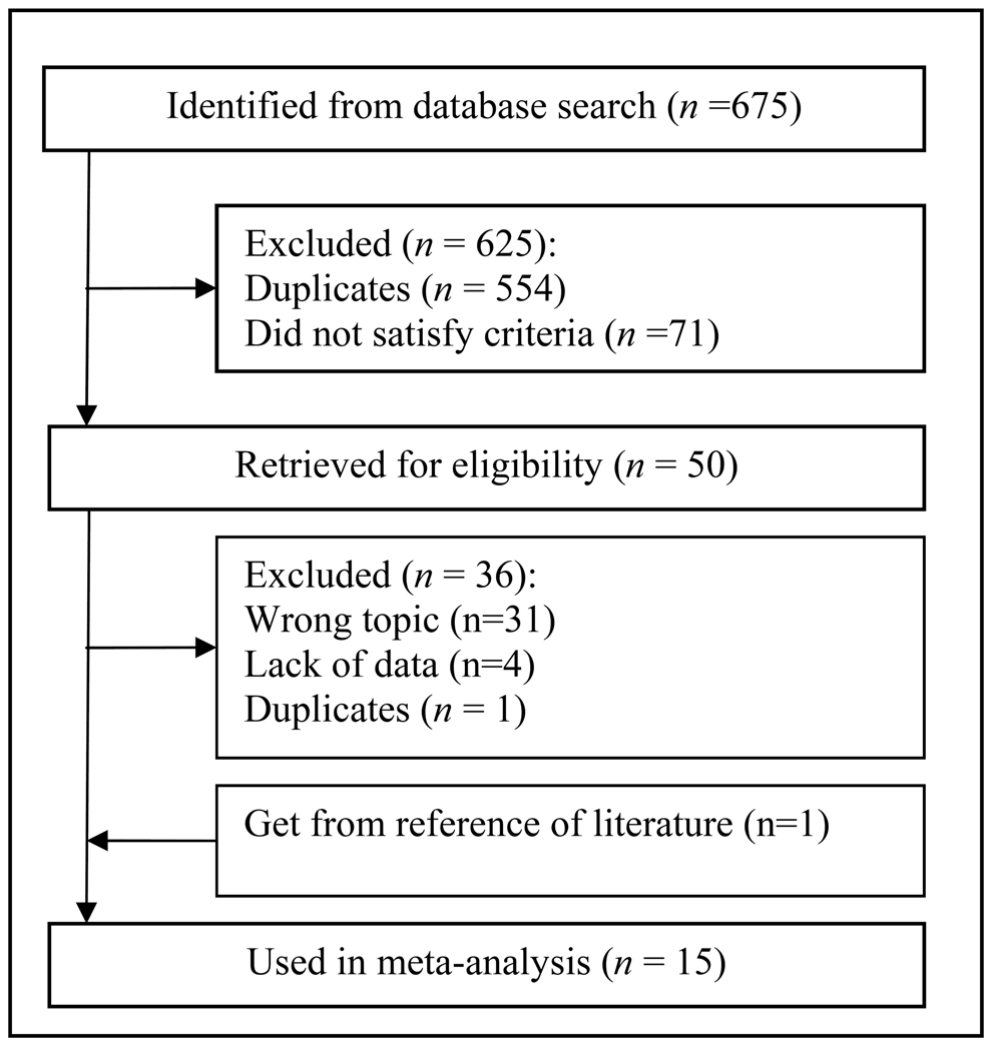
Flowchart for study selection.

The database search yielded 675 primary studies. Of these, 625 were excluded after reviewing the title and abstract, and 36 were excluded after reviewing the full article. An additional study was obtained from a review article [20]. Thus, 15 articles that included a total of 2787 patients fulfilled all the inclusion criteria and were considered in the analysis [5]–[8], [20]–[28] (Figure.1). The observers reached agreement on which studies should be included (Cohen’s unweighted κ = 0.89).

### Study Description and Quality

We identified 15 studies in which SF-WCC and 14 studies in which SF-PMN was obtained; all these studies met the eligibility criteria. Table 1 lists the included studies and describes the baseline patient characteristics. The studies were from 5 different countries (11 from the United States and 1 study each from Canada, Sweden, Korea, and United Kingdom). The median number of patients per study was 96 (range, 54–871). The median age of the research participants was 67 years (range, 64.2–71.5). A total of 8 studies prospectively enrolled patients and 7 studies were retrospective database reviews. Patient recruitment was consecutive in 7 studies and was not documented in the other 8. Only 9 of the 15 studies excluded inflammatory arthropathy. Four studies detected PJI on the hip and knee, 4 detected PJI on the hip, and 7 on the knee. The QUADAS quality assessment tool was used to evaluate each selected study. All the eligible studies scored >9 points indicating that they were of moderate quality.

**Table 1:** Characteristics of the 15 studies in our meta-analysis of the diagnosis of PJI using SF-WCC and SF-PMN.

Study	Country	Patients number	Meanage (y)	Study design, Enrollment	Sample obtain	Excluded inflammatory arthropathy	Sample type	Cut-off	Sample part	Ref Standard
Dinneen et al, 2013	United Kingdom	75	70.3	Prospective, NA	Preoperatively	Yes	SF WCC;SF PMN	1590/µl; 65%	Hip or Knee	IOF, H, M
Cipriano et al, 2012	United States	871	65	Prospective, Consecutive	Preoperatively or Intraoperatively	Yes	SF WCC; SF PMN	3450/µl; 78%	Hip or Knee	IOF, H, M
Schwartz et al, 2012	United States	96	65	Retrospective, Consecutive	NA	No	SF WCC; SF PMN	6200/µL; 60%	Knee	IOF, H, M
Kusuma et al, 2011	United States	76	65	Retrospective, Consecutive	Preoperatively	NA	SF WCC; SF PMN	1102.5/µL; 71.5%	Knee	IOF, H
Shukla et al, 2010	United States	86	64.2	Retrospective, Consecutive	Intraoperatively	No	SF WCC; SF PMN	3528/µL; 79%	Hip	IOF, H, M
Lee et al, 2010	Korea	56	69.6	Retrospective, NA	Preoperatively	Yes	SF WCC; SF PMN	3800/uL; 89%	Knee	IOF, H, M
Schinsky et al, 2008	United States	220	64.9	Prospective, Consecutive	Intraoperatively	Yes	SF WCC; SF PMN	4200/mL; 80%	Hip	M, H
Ghanem et al, 2008	United States	429	67	Retrospective, NA	Preoperatively	Yes	SF WCC; SF PMN;SF PMN	1100/uL; 64%; 73%	Knee	IOF, H, M
Trampuz et al, 2007	United States	160	69	Prospective, NA	Preoperatively	No	SF WCC; SF PMN	1700/µl; 65%	Hip or Knee	IOF, H
Nilsdotter-Augustinsson et al, 2007	Sweden	54	71.5	Prospective, Consecutive	Preoperatively	Yes	SF WCC	1700/µl	Hip	M
Della Valle et al, 2007	United States	94	66.6	Retrospective, Consecutive	Preoperatively	NA	SF WCC; SF PMN	3000/mL; 65%	Knee	IOF, H, M
Parvizi et al, 2006	United States	168	68	Prospective, NA	Intraoperatively	Yes	SF WCC; SF PMN	1760/µL; 73%	Hip or Knee	IOF, M
Trampuz et al, 2004	United States	133	71	Prospective, NA	Preoperatively	Yes	SF WCC; SF PMN	1700/µl; 65%	Knee	IOF, H, M
Mason et al, 2003	United States	86	NA	Retrospective, NA	Preoperatively	No	SF WCC; SF PMN	2500/ml; 60%	Knee	H, M
Spangehl et al, 1999	Canada	183	65	Prospective, NA	Intraoperatively	Yes	SF WCC; SF PMN	50000/µl; 80%	Hip	IOF, H, M

H: histological examination; IOF: Intraoperative finding; M: microbiological or laboratory examination; NA, not available; SF:Synovial fluid PMN: polymorphonuclear leukocytes; SF: synovial fluid; WCC: white cell count.

### Diagnostic Accuracy

**Figure 2:**
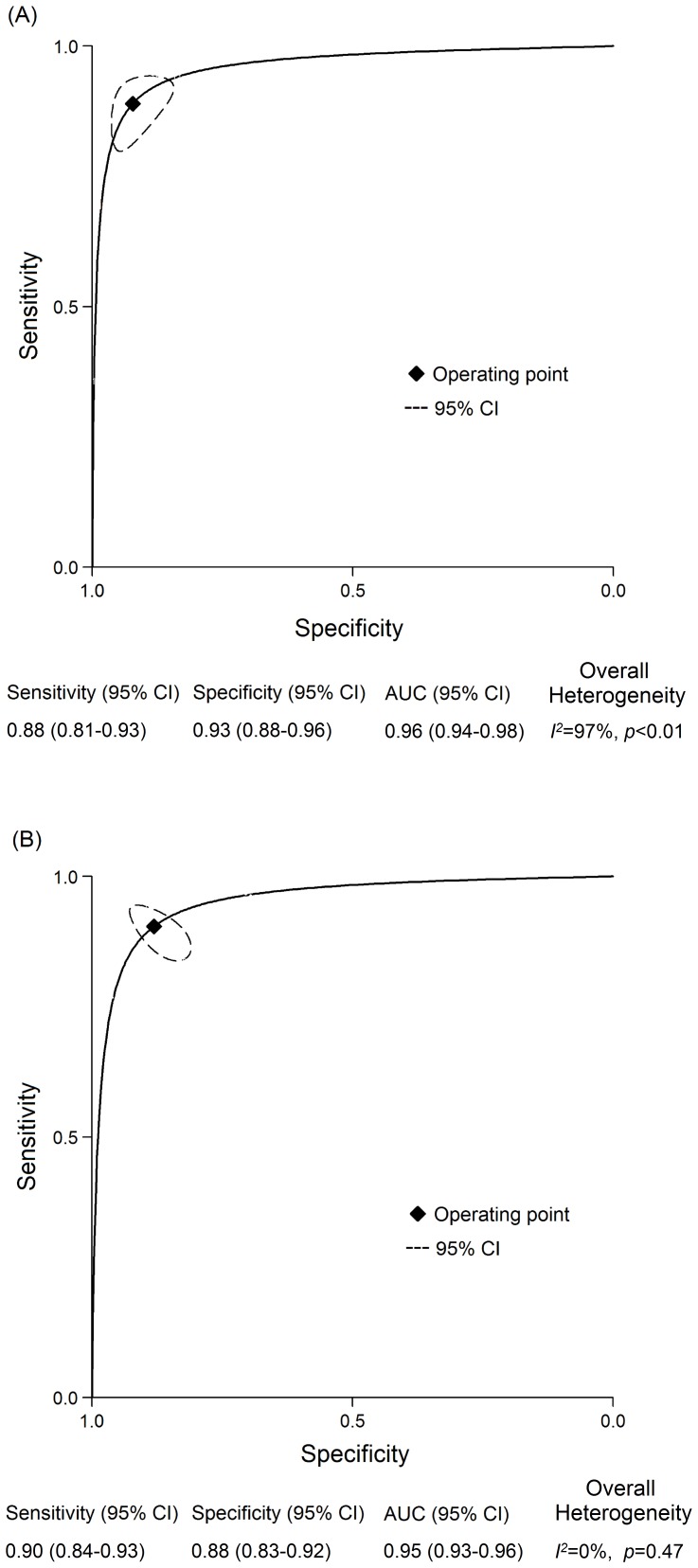
Summary receiver-operating characteristic curves for SF-WCC (A) and SF-PMN (B). Curves include a summary operating point for sensitivity and specificity on the curve and a 95% confidence contour ellipsoid.

**Figure 3:**
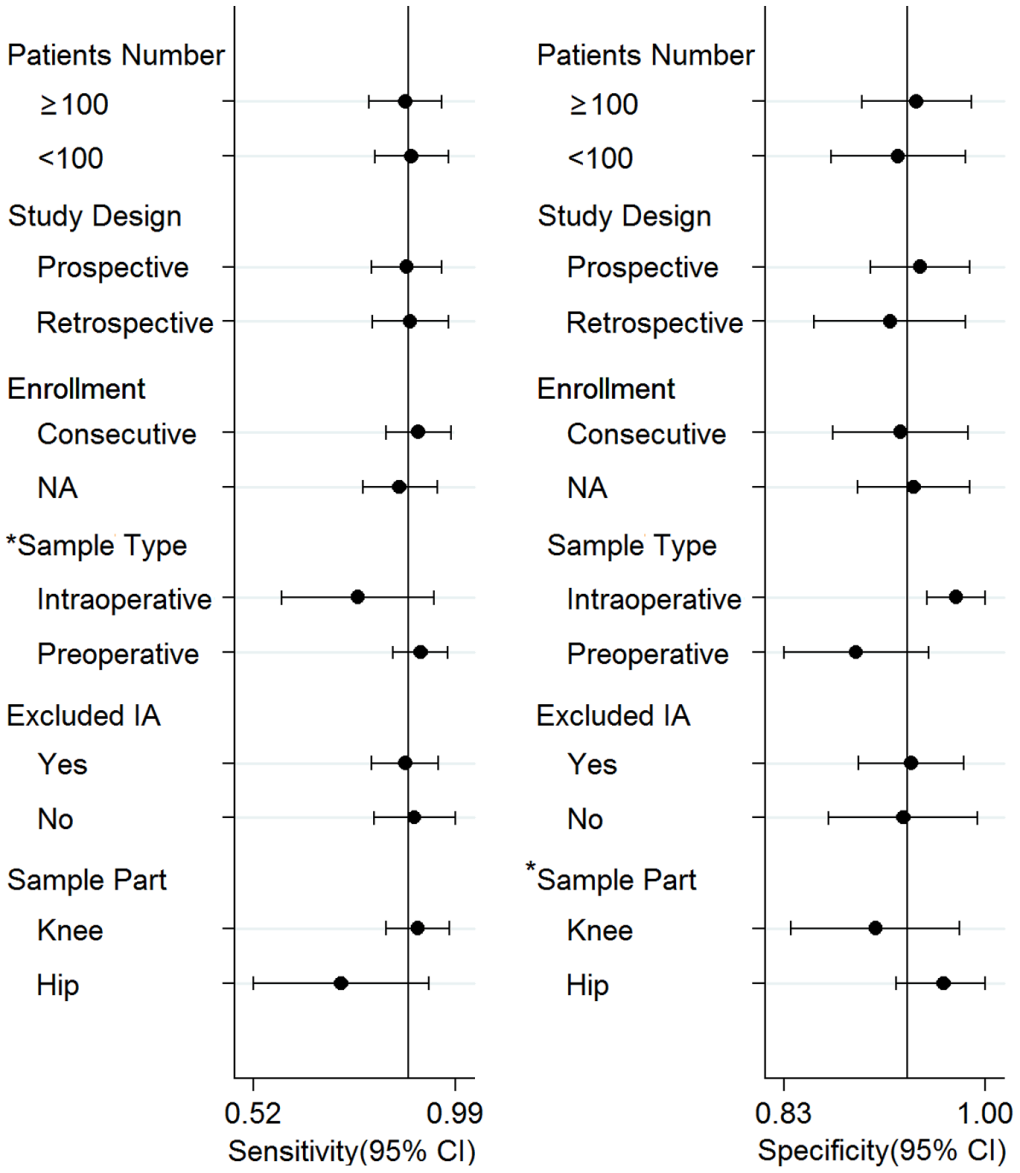
Meta-regression analyses of the sensitivity and specificity of SF-WCC.

The pooled sensitivity, specificity, DOR, and AUC obtained from the random effects model are shown in Figure. 2. The pooled sensitivity for the detection of PJI using SF-WCC and SF-PMN values were 0.88 (95% CI, 0.81–0.93) and 0.90 (95% CI, 0.84–0.93), respectively. The pooled specificity for the detection of PJI using SF-WCC and SF-PMN values were 0.93 (95% CI, 0.88–0.96) and 0.88 (95% CI, 0.83–0.92), respectively. The pooled DORs were 103 (95% CI, 54–197) for SF-WCC and 64 (95% CI, 27–149) for SF-PMN. The pooled AUC for SF-WCC and SF-PMN values were 0.96 (95% CI, 0.94–0.98) and 0.95 (95% CI, 0.93–0.96), respectively. The inconsistency index indicated that no heterogeneity was found with respect to SF-PMN (*I* ^2^ = 0%, p = 0.47). In contrast, the inconsistency index for the overall heterogeneity of SF-WCC was 97% (p<0.01), which was considered to indicate significant heterogeneity. Therefore, meta-regression analysis was subsequently performed to explore potential sources of heterogeneity in the SF-WCC studies (Figure. 3). The analyses on both the sensitivity and specificity for the detection of PJI using SF-WCC indicated no influence of the number of patients (≥100 vs. <100), study design (perspective vs. retrospective), patient enrollment (consecutive vs. not available), or exclusion of inflammatory arthropathy (yes vs. no). In contrast, we found that the contribution to the heterogeneity origin was the time at which the sample was obtained (preoperative vs. intraoperative) for sensitivity and the operative site (THA vs. TKA) for specificity (all p<0.05).

### Evaluation of Clinical Utility

**Figure 4:**
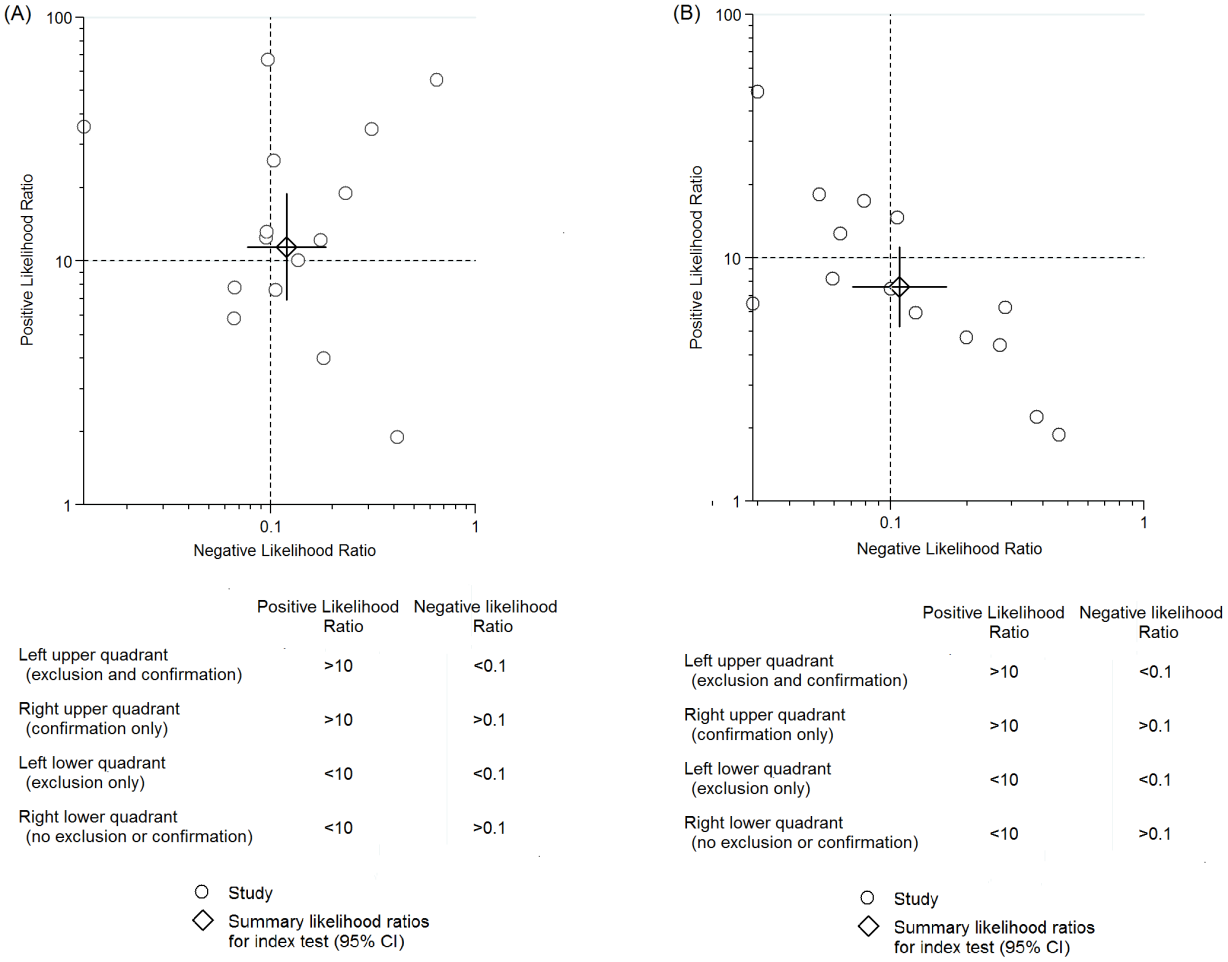
Likelihoor ratio scattergram for SF-WCC(A) and SF-PMN(B).

**Figure 5:**
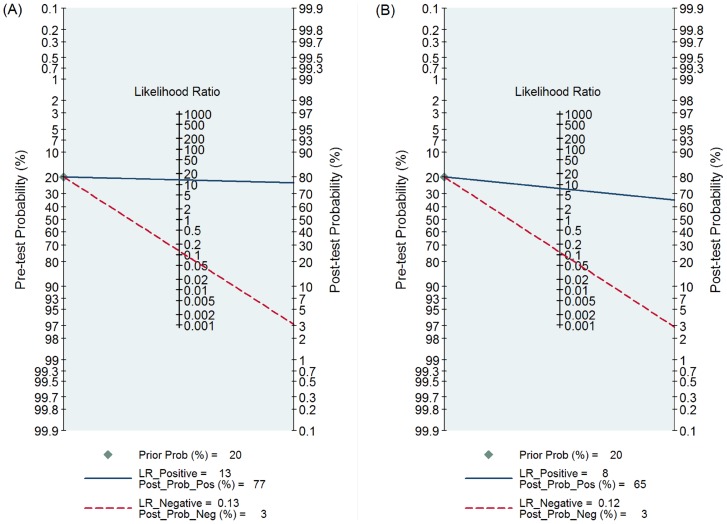
Pre-test probabilities and likelihood ratios for SF-WCC (A) and SF-PMN (B). With a pre-test probability of PJI of 20% (low clinical suspicion), the post-test probability of PJI, given a negative SF-WCC or SF-PMN result, is both 3%, which can be considered sufficient to rule out PJI.

The PLR and NLR for the diagnosis of PJI were 13.3 (95% CI, 7.7–22.8) and 0.13 (95% CI, 0.08–0.21) for SF-WCC, respectively. The PLR was 7.6 (95% CI, 4.9–11.7) and NLR was 0.12 (95% CI, 0.07–0.19) for SF-PMN (Figure. 4). We used the likelihood ratios to simulate low clinical scenarios by using 20% pre-test probabilities of PJI, and further calculated and plotted post-test probability on Fagan nomograms (Figure. 5). The post-test probability of PJI was 3%, given both negative SF-WCC or SF-PMN results, which could be considered sufficient to rule out PJI.

### Subgroup Analysis

As mentioned above, we performed a subgroup analyses on variables that were decided a priori (Table 2). The sensitivity and specificity of THA were 0.73 (95% CI, 0.56–0.85) and 0.96 (95% CI, 0.93–0.98) for SF-WCC and 0.85 (95% CI, 0.79–0.89) and 0.83 (95% CI, 0.80–0.86) for SF-PMN, respectively. The sensitivity and specificity of TKA were 0.90 (95% CI, 0.78–0.96) and 0.91 (95% CI 0.80–0.96) for SF-WCC and 0.90 (95% CI, 0.78–0.95) and 0.88 (95% CI, 0.77–0.95) for SF-PMN, respectively. The analysis also indicated that collection of SF-WCC preoperatively had a higher sensitivity than intraoperative collection of SF-WCC (0.91 vs. 0.77, p<0.05). However, compared with intraoperative SF-WCC (0.97; 95% CI, 0.93–0.99), preoperative collection of SF-WCC had a non-significant lower specificity of 0.89 (95% CI, 0.81–0.94) (p>0.05). For SF-PMN, the sensitivity and specificity of studies that excluded inflammatory arthropathy were 0.91 (95% CI, 0.85–0.95) and 0.90 (95% CI, 0.82–0.94), respectively. The studies that did not exclude inflammatory arthropathy demonstrated a sensitivity of 0.88 (95% CI, 0.75–0.95) and a specificity of 0.86 (95% CI, 0.78–0.92).

**Table 2:** Subgroup analyses for diagnosing of PJI using SF-WCC and SF-PMN.

	Number of Studies	Number of Patients	Sensitivity (95% CI)	Specificity (95% CI)	Area Under theCurve (95% CI)	Positive Likelihood Ratio (95% CI)	Negative Likelihood Ratio(95% CI)	Diagnostic Odds Ratio
**SF-WCC**								
**Overall Studies**	15	2,700	0.88 (0.81,0.93)	0.93(0.88,0.96)	0.96 (0.94,0.98)	13.3 (7.7,22.8)	0.13 (0.08,0.21)	103 (54,197)
**Number of Patients**								
<100	8	623	0.88 (0.76,0.94)	0.93 (0.84,0.97)	0.96 (0.94,0.97)	12.3 (5.2,29.2)	0.13 (0.06,0.28)	94 (24,367)
≥100	7	2,077	0.88 (0.75,0.94)	0.94 (0.87,0.97)	0.97 (0.95,0.98)	13.7 (7.3,25.6)	0.13 (0.07,0.26)	104 (67,161)
**Study Design**								
Prospective	8	1,777	0.89 (0.77,0.95)	0.94 (0.89,0.97)	0.97 (0.95,0.98)	14.4 (8.7,23.8)	0.12 (0.06,0.24)	120 (75,193)
Retrospective	7	923	0.88 (0.76,0.94)	0.92 (0.82,0.97)	0.96 (0.93,0.97)	11.5 (4.4,30.0)	0.13 (0.06,0.28)	88 (20,383)
**Patients Enrollment**								
Consecutive	7	1,410	0.90 (0.84,0.93)	0.93 (0.88,0.96)	0.96 (0.94,0.98)	13.2 (7.1,24.5)	0.11 (0.07,0.18)	120 (41,345)
Not document	8	1,290	0.87 (0.75,0.94)	0.94 (0.86,0.97)	0.96 (0.94,0.98)	13.7 (6.4,29.2)	0.14 (0.07,0.27)	98 (54,175)
**Sample obtain**								
Preoperative	9	1,163	0.91 (0.82,0.95)	0.89 (0.81,0.94)	0.95 (0.93,0.97)	8.2 (4.5,14.8)	0.11 (0.06,0.21)	75 (26,216)
Intraoperative	4	638	0.77 (0.51,0.91)	0.97 (0.93,0.99)	0.97 (0.96,0.98)	27.8 (11.5,67.3)	0.24 (0.10,0.56)	116 (41,333)
**Excluded inflammatory arthropathy**								
Yes	9	2,102	0.88 (0.79,0.93)	0.93 (0.88,0.96)	0.96 (0.94,0.98)	13.0 (7.7,21.9)	0.13 (0.07,0.23)	100 (65,153)
No	6	598	0.89 (0.72,0.97)	0.94 (0.82,0.98)	0.97 (0.95,0.98)	13.8 (4.5,42.2)	0.11 (0.04,0.34)	122 (20,740)
**Sample part**								
Hip	4	524	0.73 (0.56,0.85)	0.96 (0.93,0.98)	0.96 (0.93,0.97)	19.1 (11.1,33.0)	0.28 (0.16,0.48)	68 (39,117)
Knee	7	970	0.90 (0.78,0.96)	0.91 (0.80,0.96)	0.96 (0.94,0.97)	9.8 (4.1,23.3)	0.11 (0.05,0.26)	88 (21,381)
**SF-PMN**								
**Overall Studies**	14	2,726	0.90 (0.84,0.93)	0.88 (0.83,0.92)	0.95 (0.93,0.96)	7.6 (4.9,11.7)	0.12(0.07,0.19)	64 (27,149)
**Number of Patients**								
<100	7	564	0.86 (0.74,0.92)	0.83 (0.73,0.89)	0.91 (0.88,0.93)	5.0 (3.0,8.3)	0.17 (0.09,0.34)	28 (9,85)
≥100	7	2,162	0.93 (0.88,0.96)	0.92 (0.87,0.95)	0.97 (0.95,0.98)	11.3 (6.8,19.2)	0.08 (0.05,0.14)	141 (53,371)
**Study Design**								
Prospective	7	1,808	0.92 (0.88,0.94)	0.91 (0.87,0.94)	0.96 (0.94,0.98)	10.0 (7.0,14.3)	0.09 (0.06,0.13)	108 (56,209)
Retrospective	7	918	0.87 (0.75,0.93)	0.85 (0.74,0.91)	0.92 (0.90,0.94)	5.7 (3.1,10.7)	0.16 (0.07,0.27)	36 (10,132)
**Patients Enrollment**								
Consecutive	6	1,438	0.89 (0.83,0.94)	0.84 (0.79,0.89)	0.93 (0.90,0.95)	5.7 (4.1,8.1)	0.13 (0.07,0.22)	46 (20,106)
Not Available	8	1,288	0.90 (0.85,0.94)	0.90 (0.86,0.94)	0.96 (0.94,0.97)	9.5 (6.0,14.8)	0.11 (0.07,0.17)	87 (37,205)
**Sample obtain**								
Preoperative	8	1,109	0.90 (0.80,0.95)	0.88 (0.78,0.94)	0.95 (0.93,0.97)	7.6 (3.8,15.1)	0.11 (0.05,0.24)	66 (16,267)
Intraoperative	4	655	0.88 (0.80,0.93)	0.86 (0.80,0.90)	0.93 (0.91,0.95)	6.3 (4.2,9.4)	0.14 (0.08,0.24)	44 (19,103)
**Excluded inflammatory arthropathy**								
Yes	8	2,133	0.91 (0.85,0.95)	0.90 (0.82,0.94)	0.96 (0.94,0.97)	8.7 (4.7,16.3)	0.10 (0.06,0.18)	85 (27,271)
No	6	593	0.88 (0.75,0.95)	0.86 (0.78,0.92)	0.93 (0.91,0.95)	6.4 (3.7,11.0)	0.14 (0.06,0.32)	46 (13,158)
**Sample part**								
Hip	3	487	0.85 (0.79,0.89)	0.83 (0.80,0.86)	0.91 (0.88,0.93)	5.1 (4.3,6.0)	0.18 (0.13,0.26)	28 (18,44)
Knee	7	965	0.90 (0.78,0.95)	0.88 (0.77,0.95)	0.95 (0.93,0.97)	7.7 (3.4,17.3)	0.12 (0.05,0.28)	66(13,325)

NA, not available; PMN: polymorphonuclear leukocytes; SF: synovial fluid; WCC: white cell count.

### Assessment of Publication Bias

**Figure 6:**
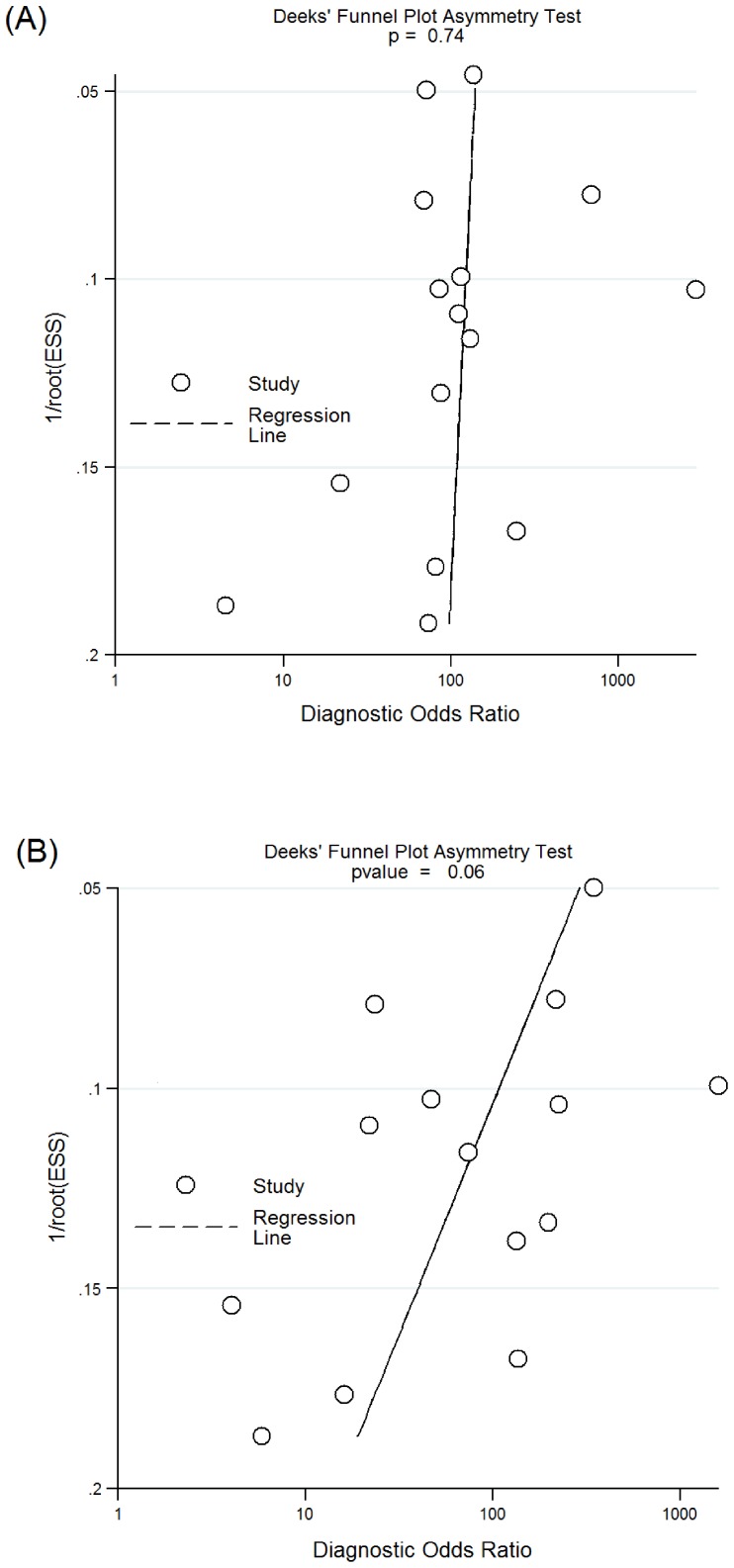
Funnel plots for included studies. (A) SF-WCC; (B) SF-PMN.

To assess for potential publication bias, Deeks’ funnel plots were created by plotting the logDOR of the individual studies against their sample size. The funnel plots for SF-WCC and SF-PMN are presented in Figure 6. The regression test of asymmetry found no evidence of a small-study effect for either SF-WCC (p = 0.74) or SF-PMN (p = 0.06).

## Discussion

In this meta-analysis of 15 articles with a total of 2787 patients, we found that SF-WCC and SF-PMN could be used for distinguishing among PJIs among patients who underwent THA or TKA. The high sensitivity, specificity, and AUC demonstrated a high diagnostic accuracy of SF-WCC and SF-PMN. Furthermore, the PLR and NLR findings, as well low-clinical-scenarios post-test probabilities illustrate the clinical applicability SF-WCC and SF-PMN. We also found that preoperative aspiration of SF-WCC had a higher sensitivity than intraoperative aspiration and SF-PMN had a higher sensitivity for TKA, compared to THA. Lastly, studies that excluded inflammatory arthropathy had a non-significant higher sensitivity and specificity than the studies did not exclude of inflammatory arthropathy. Collectively, these meta-analysis findings demonstrate the clinical utility of SF-WCC and SF-PMN to accurately diagnose PJI after TKA or THA.

The diagnosis of PJI after THA or TKA remains a challenge, for which many preoperative and intraoperative tests have been employed. Unfortunately, none of current tests has perfect sensitivity and specificity [1], [2]. Over the past decade, many studies have reported that fluorodeoxyglucose-positronemission tomography (FDG-PET) and antigranulocyte scintigraphy with ^99m^Tc-labeled monoclonal antibodies are good imaging modalities for PJI diagnosis. Two meta-analyses demonstrated acceptable diagnostic capability and indicated that the sensitivity of FDG-PET and antigranulocyte scintigraphy were 0.82 and 0.83, and the specificity was 0.87 and 0.80, respectively [29], [30]. However, the expensive cost, complex techniques, and the requirement for special operators limit the clinical application of these diagnostic techniques. White blood cell (WBC) count, erythrocyte sedimentation rate (ESR), and C-reactive protein (CRP) are the most common preoperative laboratory tests used for the diagnosis of PJI [2], [3], [10]. However, a meta-analysis performed by Berbari et al. [31] showed that the diagnostic ability of these laboratory tests are not entirely reliable. Indeed, the accuracy of inflammation markers, represented with DORs, was 13.1 for CRP, 7.2 for ESR, and 4.4 for WBC.

Guidelines by AAOS and IDSA strongly recommend that patient’s SF-WCC and SF-PMN be assessed for PJI [10]–[12]. Consistent with the AAOS and IDSA guidelines, our results show that SF-WCC and SF-PMN are diagnostic methods that have both a high sensitivity and specificity. However, the true diagnostic ability of these tests depends on whether the synovial fluid aspiration is successful. Many factors can influence the final result, such as synovial fluid volume or antibiotic use. In clinical, when the preoperative serum inflammation markers are above the threshold for PJI in the absence of a known cause, further aspiration of the joint is warranted [10]. Detection of SF-WCC and SF-PMN was the second step recommended by the AAOS guidelines, and it is inexpensive. [12] In addition, we must highlight that with a joint aspiration sample, culture also can be realized. Another meta-analysis evaluated preoperative aspiration culture for diagnosing PJI and found that preoperative aspiration culture has moderate to high sensitivity at 0.72 (95% CI, 0.65–0.78) and very high specificity at 0.95 (95%CI, 0.93–0.97) for diagnosing PJI [32]. Furthermore, low-grade infections caused by low-virulent microorganisms usually have normal values of SF-WCC and SF-PMN [32]. So it is important to performing preoperative aspiration culture if there is a high suspicion of PJI although values of SF-WCC and SF-PMN are normal [32]. In addition, preoperative aspiration culture may identify a pathogen for making treatment plan.

Moreover, there is little consensus regarding the cut-off values for SF-WCC or SF-PMN. In our meta-analysis, the cut-off values ranged from 2500 to 50000/µL for SF-WCC and 60–89% for SF-PMN. The workgroup convened by the Musculoskeletal Infection Society acknowledged that the cut-off level for SF-WCC or SF-PMN has not been well delineated [33]. However, due to different patient characteristics in the individual studies, it is difficult to determine the optimal cut-off values in the current study. Additional patient-level meta-analyses are required to reliably address this issue.

There are several limitations to the current study. First, there is no established gold standard for diagnosing PJI. In our meta-analysis, many reference standards were used in the individual studies, including clinical manifestation (purulence or fistula), laboratory studies (acute inflammation in histopathology or in blood) and microbiological growth (in periprosthetic tissues or in sonication fluid culture). None of these methods is perfect as a reference standard for diagnosing PJI. Misclassification bias, resulting from an imperfect reference standard, may affect the estimates of diagnostic accuracy of a tested method [29]. In general, this leads to an underestimation of the diagnostic accuracy.

Second, the summary results of SF-WCC had high statistical heterogeneity. Therefore, we performed a thorough meta-regression analysis to investigate possible sources of heterogeneity. We found that the time at which the sample was obtained and the operative site contributed to the heterogeneity origin for sensitivity and specificity, respectively. This issue may reduce the strength of the conclusions that can be drawn from this meta-analysis for SF-WCC. Moreover, due to absence of stratified data, it is hard to perform a subgroup analyses for race, gender or age, which may influence the accuracy in diagnosing PJI. Future studies are needed to certify this affection.

Third, not all the studies that were examined explicitly stated whether they were performed in a prospective manner. However, including a prospective study design such as a covariate to the bivariate statistical model (prospective design vs. retrospective design) did not significantly influence sensitivity or specificity.

Fourth, only a few studies reported the use of antibiotics or the time between the assessment of synovial fluid analysis and the validation of PJI. This may affect the diagnosis accuracy. Furthermore, various cut-off values were used in the individual studies. However, it is difficult to determine the best cut-off value of these tests. The use of antibiotics may lead to increased false negatives, and the presence of inflammatory arthropathy may induce false positives.

In summary, this diagnostic accuracy meta-analysis demonstrates that SF-WCC and SF-PMN have adequate and clinically acceptable diagnostic values for the detection of PJI, particularly after TKA. Our results are consistent with the AAOS and IDSA guidelines although the optimal cut-off values of these tests may need further large-scale validation.

## References

[1] Clohisy JC, Calvert G, Tull F, McDonald D, Maloney WJ (2004) Reasons for revision hip surgery: a retrospective review. Clin Orthop Relat Res: 188–192. 10.1097/01.blo.0000150126.73024.42 15577486

[2] Del Pozo JL, Patel R (2009) Clinical practice. Infection associated with prosthetic joints. N Engl J Med 361: 787–794. 19692690 10.1056/NEJMcp0905029 PMC2850113

[3] Trampuz A, Piper KE, Jacobson MJ, Hanssen AD, Unni KK, et al (2007) Sonication of removed hip and knee prostheses for diagnosis of infection. N Engl J Med 357: 654–663. 17699815 10.1056/NEJMoa061588

[4] Patel R, Osmon DR, Hanssen AD (2005) The diagnosis of prosthetic joint infection: current techniques and emerging technologies. Clin Orthop Relat Res: 55–58. 10.1097/01.blo.0000175121.73675.fd 16056026

[5] Trampuz A, Hanssen AD, Osmon DR, Mandrekar J, Steckelberg JM, et al (2004) Synovial fluid leukocyte count and differential for the diagnosis of prosthetic knee infection. Am J Med 117: 556–562. 15465503 10.1016/j.amjmed.2004.06.022

[6] Ghanem E, Parvizi J, Burnett RS, Sharkey PF, Keshavarzi N, et al (2008) Cell count and differential of aspirated fluid in the diagnosis of infection at the site of total knee arthroplasty. J Bone Joint Surg Am 90: 1637–1643. 18676892 10.2106/JBJS.G.00470

[7] Cipriano CA, Brown NM, Michael AM, Moric M, Sporer SM, et al (2012) Serum and synovial fluid analysis for diagnosing chronic periprosthetic infection in patients with inflammatory arthritis. J Bone Joint Surg Am 94: 594–600. 22488615 10.2106/JBJS.J.01318

[8] Dinneen A, Guyot A, Clements J, Bradley N (2013) Synovial fluid white cell and differential count in the diagnosis or exclusion of prosthetic joint infection. Bone Joint J 95-B: 554–557. 23539710 10.1302/0301-620X.95B4.30388

[9] Qu X, Zhai Z, Li H, Li H, Liu X, et al (2013) PCR-based diagnosis of prosthetic joint infection. J Clin Microbiol 51: 2742–2746. 23740731 10.1128/JCM.00657-13 PMC3719604

[10] Parvizi J, Adeli B, Zmistowski B, Restrepo C, Greenwald AS (2012) Management of periprosthetic joint infection: the current knowledge: AAOS exhibit selection. J Bone Joint Surg Am 94: e104. 22810411 10.2106/JBJS.K.01417

[11] Osmon DR, Berbari EF, Berendt AR, Lew D, Zimmerli W, et al (2013) Executive summary: diagnosis and management of prosthetic joint infection: clinical practice guidelines by the Infectious Diseases Society of America. Clin Infect Dis 56: 1–10. 23230301 10.1093/cid/cis966

[12] Della Valle C, Parvizi J, Bauer TW, DiCesare PE, Evans RP, et al (2011) American Academy of Orthopaedic Surgeons clinical practice guideline on: the diagnosis of periprosthetic joint infections of the hip and knee. J Bone Joint Surg Am 93: 1355–1357. 21792503 10.2106/JBJS.9314ebo

[13] Deville WL, Buntinx F, Bouter LM, Montori VM, de Vet HC, et al (2002) Conducting systematic reviews of diagnostic studies: didactic guidelines. BMC Med Res Methodol 2: 9. 12097142 10.1186/1471-2288-2-9 PMC117243

[14] Liberati A, Altman DG, Tetzlaff J, Mulrow C, Gotzsche PC, et al (2009) The PRISMA statement for reporting systematic reviews and meta-analyses of studies that evaluate health care interventions: explanation and elaboration. Ann Intern Med 151: W65–94. 19622512 10.7326/0003-4819-151-4-200908180-00136

[15] Berlin JA (1997) Does blinding of readers affect the results of meta-analyses? University of Pennsylvania Meta-analysis Blinding Study Group. Lancet 350: 185–186. 10.1016/s0140-6736(05)62352-5 9250191

[16] Whiting P, Rutjes AW, Reitsma JB, Bossuyt PM, Kleijnen J (2003) The development of QUADAS: a tool for the quality assessment of studies of diagnostic accuracy included in systematic reviews. BMC Med Res Methodol 3: 25. 14606960 10.1186/1471-2288-3-25 PMC305345

[17] Moses LE, Shapiro D, Littenberg B (1993) Combining independent studies of a diagnostic test into a summary ROC curve: data-analytic approaches and some additional considerations. Stat Med 12: 1293–1316. 8210827 10.1002/sim.4780121403

[18] Huedo-Medina TB, Sanchez-Meca J, Marin-Martinez F, Botella J (2006) Assessing heterogeneity in meta-analysis: Q statistic or I2 index? Psychol Methods 11: 193–206. 16784338 10.1037/1082-989X.11.2.193

[19] Deeks JJ, Macaskill P, Irwig L (2005) The performance of tests of publication bias and other sample size effects in systematic reviews of diagnostic test accuracy was assessed. J Clin Epidemiol 58: 882–893. 16085191 10.1016/j.jclinepi.2005.01.016

[20] Parvizi J, Ghanem E, Menashe S, Barrack RL, Bauer TW (2006) Periprosthetic infection: what are the diagnostic challenges? J Bone Joint Surg Am 88 Suppl 4138–147. 10.2106/JBJS.F.00609 17142443

[21] Della Valle CJ, Sporer SM, Jacobs JJ, Berger RA, Rosenberg AG, et al (2007) Preoperative testing for sepsis before revision total knee arthroplasty. J Arthroplasty 22: 90–93. 17823024 10.1016/j.arth.2007.04.013

[22] Lee SC, Jung KA, Yoon JY, Nam CH, Hwang SH, et al (2010) Analysis of synovial fluid in culture-negative samples of suspicious periprosthetic infections. Orthopedics 33: 725. 20954662 10.3928/01477447-20100826-13

[23] Schinsky MF, Della Valle CJ, Sporer SM, Paprosky WG (2008) Perioperative testing for joint infection in patients undergoing revision total hip arthroplasty. J Bone Joint Surg Am 90: 1869–1875. 18762646 10.2106/JBJS.G.01255

[24] Spangehl MJ, Masri BA, O’Connell JX, Duncan CP (1999) Prospective analysis of preoperative and intraoperative investigations for the diagnosis of infection at the sites of two hundred and two revision total hip arthroplasties. J Bone Joint Surg Am 81: 672–683. 10360695 10.2106/00004623-199905000-00008

[25] Mason JB, Fehring TK, Odum SM, Griffin WL, Nussman DS (2003) The value of white blood cell counts before revision total knee arthroplasty. J Arthroplasty 18: 1038–1043. 14658109 10.1016/s0883-5403(03)00448-0

[26] Nilsdotter-Augustinsson A, Briheim G, Herder A, Ljunghusen O, Wahlstrom O, et al (2007) Inflammatory response in 85 patients with loosened hip prostheses: a prospective study comparing inflammatory markers in patients with aseptic and septic prosthetic loosening. Acta Orthop 78: 629–639. 17966022 10.1080/17453670710014329

[27] Education SoURaC (2012) Diagnosis of periprosthetic joint infection after unicompartmental knee arthroplasty. J Arthroplasty 27: 46–50. 22841381 10.1016/j.arth.2012.03.033

[28] Shukla SK, Ward JP, Jacofsky MC, Sporer SM, Paprosky WG, et al (2010) Perioperative testing for persistent sepsis following resection arthroplasty of the hip for periprosthetic infection. J Arthroplasty 25: 87–91. 10.1016/j.arth.2010.05.006 20732621

[29] Pakos EE, Trikalinos TA, Fotopoulos AD, Ioannidis JP (2007) Prosthesis infection: diagnosis after total joint arthroplasty with antigranulocyte scintigraphy with 99mTc-labeled monoclonal antibodies–a meta-analysis. Radiology 242: 101–108. 17090716 10.1148/radiol.2421052011

[30] Kwee TC, Kwee RM, Alavi A (2008) FDG-PET for diagnosing prosthetic joint infection: systematic review and metaanalysis. Eur J Nucl Med Mol Imaging 35: 2122–2132. 18704405 10.1007/s00259-008-0887-x

[31] Berbari E, Mabry T, Tsaras G, Spangehl M, Erwin PJ, et al (2010) Inflammatory blood laboratory levels as markers of prosthetic joint infection: a systematic review and meta-analysis. J Bone Joint Surg Am 92: 2102–2109. 20810860 10.2106/JBJS.I.01199

[32] Qu X, Zhai Z, Wu C, Jin F, Li H, et al (2013) Preoperative aspiration culture for preoperative diagnosis of infection in total hip or knee arthroplasty. J Clin Microbiol 51: 3830–3834. 23946521 10.1128/JCM.01467-13 PMC3889774

[33] Workgroup Convened by the Musculoskeletal Infection S (2011) New definition for periprosthetic joint infection. J Arthroplasty 26: 1136–1138. 22075161 10.1016/j.arth.2011.09.026

